# Venous Thromboembolism in Severe Community‐Acquired Pneumonia at High‐Altitude Areas: Prevalence, Risk and Outcome

**DOI:** 10.1111/crj.70230

**Published:** 2026-09-10

**Authors:** Yuntao Zhang, Yanyan Zhang, Basang Xiao, Miao Lu, Jianghe Jia, Xingya Shi, Xiwangmu Yi, Yuanhua Yang, Na Cui

**Affiliations:** ^1^ Department of Respiratory and Critical Care Medicine Lhasa People's Hospital Lhasa Tibet China; ^2^ Department of Respiratory and Critical Care Medicine, Beijing Institute of Respiratory Medicine and Beijing Chao‐Yang Hospital Capital Medical University Beijing China; ^3^ Department of Beijing Key Laboratory of Multimodal Intelligent Diagnosis and Treatment System for Respiratory Diseases Beijing China

**Keywords:** community‐acquired pneumonia, high‐altitude, severe, thromboprophylaxis, venous thromboembolism

## Abstract

**Introduction:**

High‐altitude exposure and hypoxia may induce a hypercoagulable state, thereby promoting the development of venous thromboembolism (VTE). Few studies have reported the incidence of VTE among patients with severe community‐acquired pneumonia (SCAP) in high‐altitude regions. We conducted a study to investigate the prevalence, risk factors, prognosis, and potential thromboprophylaxis strategies of VTE in this patient population.

**Methods:**

We prospectively enrolled 158 adult patients with SCAP admitted to Lhasa People's Hospital between January 1, 2023, and December 31, 2024. All patients underwent lower extremity venous compression ultrasound and/or computed tomography pulmonary angiography examination. Clinical characteristics, including demographic information, medical history, vital signs, laboratory findings, treatments, complications, and clinical outcomes, were compared for patients with and without VTE in these two subgroups.

**Results:**

Of the 158 patients, 74 (46.8%) developed VTE, including 68 with DVT, 21 with pulmonary embolism (PE) and 15 with both DVT and PE. A total of 123 (77.8%) patients received VTE prophylaxis, and there was no significant difference in the incidence of VTE among the groups with different prophylactic measures (*p* = 0.838). Multivariate logistic regression analysis showed an association between lower partial pressure of arterial oxygen (PaO_2_)/fraction of inspired oxygen (FiO_2_) ratio (PaO_2_/FiO_2_ ratios ≤ 160 mmHg: odds ratio [OR] = 2.354, 95% confidence interval [CI] 1.025–5.406, *p* = 0.044), higher D‐dimer level (D‐dimer levels ≥ 3.0 μg/mL: OR = 2.901, 95% CI 1.329–6.334; *p* = 0.008), longer bedridden time (bedridden times ≥ 3 days: OR = 3.540, 95% CI 1.494–8.391, *p* = 0.004), and the occurrence of VTE. A new prediction model including PaO_2_/FiO_2_ ratios, D‐dimer levels, and bedridden times showed a better performance in predicting VTE (AUC = 0.886; 95% CI 0.826–0.931; sensitivity: 74.3%; specificity: 89.3%) than Padua prediction score (AUC = 0.624) and Caprini prediction score (AUC = 0.622) for patients with SCAP at high‐altitude areas (all *p* < 0.05). The calibration curve of the nomogram model shows good predictive value. The 30‐day cumulative survival rate of patients with VTE was significantly lower than that of patients without VTE (*p* = 0.034).

**Conclusions:**

The prevalence of VTE is unexpectedly high in hospitalized patients with SCAP in high‐altitude regions and may be associated with a poor prognosis. Early identification of patients at high risk for VTE and optimization of thromboprophylactic strategies are therefore warranted improve patient outcomes.

## Graphical abstract

In hospitalized patients with SCAP at high‐altitude areas, the prevalence of VTE is unexpectedly high and may be associated with poor prognosis. Early identification of high‐risk individuals and refinement of preventive strategies are therefore necessary to improve patient outcomes.

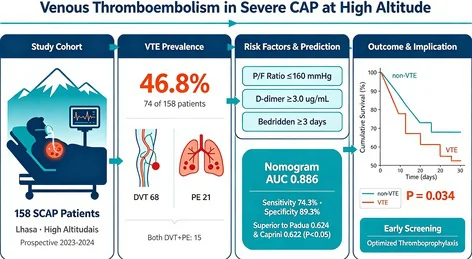

**Abbreviations**
ALT: alanine aminotransferase
APACHE: Acute Physiology and Chronic Health Evaluation
ARDS: acute respiratory distress syndrome
AUC: area under the curve
CI: confidence interval
COVID‐19: coronavirus disease 2019
CT: computerized tomography
CTPA: computed tomography pulmonary angiography
CURB‐65: confusion, blood urea nitrogen, respiratory rate, blood pressure, age ≥ 65 years
DVT: deep vein thrombosis
EF: ejection fraction
FiO_2_
: fraction of inspired oxygen
ICU: intensive care unit
IMV: invasive mechanical ventilation
IQR: interquartile range
LA: left atrial
LMWH: low molecular weight heparin
LV: left ventricular
LVEDVI: left ventricular end‐diastolic volume index
LVESVI: left ventricular end‐systolic volume index
NIV: noninvasive mechanical ventilation
OR: odds ratio
PAH: pulmonary artery hypertension
PaO_2_
: partial pressure of arterial oxygen
PASP: pulmonary artery systolic pressure
PE: pulmonary embolism
PSI: pneumonia severity index
RA: right atrial
ROC: receiver operating characteristic
RV: right ventricular
SCAP: severe community‐acquired pneumonia
SD: standard deviation
SOFA: Sequential Organ Failure Assessment
VTE: venous thromboembolism

## Introduction

Venous thromboembolism (VTE), which includes deep vein thrombosis (DVT) and pulmonary embolism (PE), is the third leading cause of vascular disease–related mortality worldwide. Its pathogenesis is associated with hypercoagulability, endothelial injury, and blood stasis. Infection and inflammation are closely intertwined with thrombosis, especially in severe infections, where the interplay of inflammation, coagulation–fibrinolysis dysregulation, endothelial damage, and thromboembolic events constitutes a critical pathophysiological process of the disease. Since 2006, multiple studies have reported that the risk of DVT and PE increases following acute respiratory infections [1], and this risk correlates positively with both infection severity and adverse clinical outcomes [2, 3, 4, 5, 6]. Community‐acquired pneumonia (CAP) refers to infectious inflammation of the pulmonary parenchyma acquired outside the hospital setting and is a common respiratory infectious disease. Severe community‐acquired pneumonia (SCAP) is characterized by poor prognosis and extremely high 30‐day mortality [7, 8, 9]. Patients with SCAP manifest severe inflammatory reactions, endothelial injury, and a hypercoagulable state. Moreover, owing to the severity of illness, these patients often require intensive care unit (ICU) admission, sedation, immobilization, central venous catheter (CVC) placement, and invasive mechanical ventilation (IMV), all of which are established risk factors for VTE, thereby significantly increasing the risk of VTE [10, 11, 12, 13]. Our previous research demonstrated that the incidence of DVT among patients with acute respiratory distress syndrome (ARDS) secondary to SCAP reached 39.6% and was associated with poor clinical outcomes [14].

Plateau areas are typically defined as regions with an altitude higher than 2500 m [15]. The unique high‐altitude, hypobaric, hypoxic, and low‐temperature environment of these areas also has an impact on the occurrence of VTE. In 2001, Anand et al. [16] performed a VTE‐related survey on 20 257 hospitalized patients; their findings demonstrated that the incidence of VTE among individuals with long‐term residence at high altitude was 30‐fold higher than that among hospitalized patients residing in plain regions during the same period. Subsequent multiple studies have pointed out that high altitude exposure and hypoxia can induce the release of inflammatory factors, vascular endothelial injury, and a hypercoagulable state, thereby promoting development VTE [17, 18, 19, 20, 21]. In recent years, the basic research of Li et al. has shown that the hypoxic and cold stress factors in plateau environments enhance the activity of thrombin and coagulation factor XIIa by inducing the expression of transferrin, thereby promoting the formation of venous thrombosis [22].

The Qinghai–Tibet Plateau is an inland plateau in Asia, representing the highest and largest in China and the world, which is also known as the “roof of the world” and the “third pole.” Sun et al. reported that the incidence of DVT among hospitalized patients in Qinghai Province (average elevation: 3500 m) on the Qinghai–Tibet Plateau was 4.6% [23], which was significantly higher than that among medical inpatients in the plain regions of China (0.3%) [2]. Lhasa is situated on the Qinghai–Tibetan Plateau, with an average elevation of 3650 m. Our prior statistical analysis revealed that the incidence of VTE among medical inpatients in Lhasa was 14.0%, which was also markedly higher than that in plain regions. However, the incidence of VTE among patients with SCAP at high altitude remains unknown. To determine the incidence of VTE, identify its associated risk factors, and evaluate its prognostic impact in patients with SCAP residing in high‐altitude regions, we performed a prospective observational study of patients with SCAP in Lhasa on the Qinghai–Tibet Plateau, with a view to enable early identification of high‐risk individuals, inform rational thromboprophylactic strategies, and ultimately improve patient outcomes.

## Methods

### Study Design and Population

We prospectively enrolled adult patients (≥ 18 years of age) with SCAP who were admitted into Department of Pulmonary and Critical Care Medicine, Lhasa People's Hospital between January 1, 2023, and December 31, 2024. All patients were enrolled consecutively and met the diagnostic criteria of SCAP according to Clinical Practice Guideline of the American Thoracic Society (ATS) and Infectious Diseases Society of America (IDSA) [24].

Exclusion criteria were as follows: active malignant tumor, stroke, acute myocardial infarction, major trauma (injury severity score > 16), lower extremity fracture, hip or knee arthroplasty, and other major surgery with a duration > 45 min within 1 month prior to admission. Patients who had already been diagnosed with VTE prior to admission and were undergoing anticoagulation, patients with a survival time less than 1 day, and patients without lower extremity venous compression ultrasound data were also excluded.

The initial ultrasound examination was performed within 1–3 days of admission. Following intensive care, repeat ultrasound was obtained if the patient's condition deteriorated (e.g., unexplained hypoxemia or cardiac insufficiency). If there was more than one ultrasound scan for a single patient, all the results were recorded. Patients were stratified into DVT and non‐DVT groups based on lower‐extremity venous compression ultrasound results. Computed tomographic pulmonary angiography (CTPA) was performed in patients with clinical suspicion of PE. Patients were divided into PE and non‐PE groups based on CTPA findings. Patients with DVT and/or PE were assigned to the VTE group, whereas patients with neither DVT nor PE were assigned to the non‐VTE group.

The study was approved by the ethics committees of the Lhasa People's Hospital (SYLL2124057) and conducted in accordance with the 1964 Declaration of Helsinki and subsequent amendments. Written informed consent was obtained from all patients or their legal guardian(s).

### Clinical Data

Medical records, including demographic characteristics, clinical history, vital signs, laboratory results, therapeutic interventions, complications, and clinical outcomes during hospitalization, were collected and analyzed for all patients. We also analyzed the survival rates of all patients within 30 days after a diagnosis of SCAP. For patients discharged within 30 days, we followed up by phone concerning their survival status after discharge.

### Ultrasound and CTPA Assessment

Bedside ultrasound examinations were performed using a portable color ultrasound scanner (CX50, Philips Medical Systems, the Netherlands, equipped with an L12‐3/S5‐1 probe). The lower extremity venous compression ultrasound was obtained from the institution's Picture Archiving and Communication System. The levels of DVT included the bilateral common femoral, deep and superficial femoral veins, the popliteal veins, and the anterior tibial, posterior tibial, peroneal, and calf muscle veins. Left ventricular and right ventricular function parameters were captured. The presence of pulmonary artery hypertension (PAH) was evaluated by adding a tricuspid regurgitation pressure gradient to the estimated right atrial pressure. The left ventricular ejection fraction was calculated using Simpson's biplane method. For patients with clinically suspected PE, a 128‐slice spiral CT (GE Revolution CT ES) was used for CTPA examination to determine the presence of PE.

### Definitions

Pneumonia was diagnosed based on clinical manifestations, laboratory test results, imaging findings and positive pathogen detections results from multiple specimens, including sputum, bronchoalveolar lavage fluid, blood, pleural effusions, and other relevant clinical samples. SCAP was defined in accordance with the ATS/IDSA Clinical Practice Guideline [24]. Distal DVT was defined as thrombosis involving the calf muscular veins or in at least one branch of the three pairs of deep calf veins (anterior tibial vein, posterior tibial vein, or peroneal vein), whereas proximal DVT was defined as thrombosis located in or above the popliteal vein or above. The diagnosis of PE via CTPA findings was established based on the following criteria: direct signs, low‐density filling defects in the pulmonary artery, partially or completely surrounded by opacified blood flow (orbital sign), or complete intraluminal filling defects, with non‐visualization of vessels visible; indirect signs comprised newly developed wedge‐shaped opacities in the lung parenchyma, banded high density area or discoid atelectasis, central pulmonary artery dilation, and diminished or absent distal vascular branches. VTE prophylactic were implemented according to the Antithrombotic Therapy and Prevention of Thrombosis, 9th edition: American College of Chest Physicians Evidence‐Based Clinical Practice Guidelines [25]. The Padua prediction score was calculated per the Barbar model [26]. The Caprini score was determined using the updated Caprini Risk Assessment Model (2013 Version) [27]. Four scoring systems were applied to evaluate disease severity: CURB‐65 (abbreviation of “confusion, blood urea nitrogen, respiratory rate, blood pressure, age ≥ 65 years”) score, pneumonia severity index (PSI) score, Acute Physiology and Chronic Health Evaluation (APACHE II) score, and Sequential Organ Failure Assessment (SOFA) score to assess the severity of disease [28, 29]. The ratio of partial pressure of arterial oxygen (PaO_2_)/fraction of inspired oxygen (FiO_2_) at high‐altitude areas was adjusted by the following formula: PaO_2_/FiO_2_ × 760/barometric pressure [30].

### Statistical Analyses

Categorical variables were described as number and percentage (%), and continuous variables were described as means ± standard deviations or medians with interquartile ranges, as appropriate. The Shapiro–Wilk test was used to evaluate if a continuous variable follows a normal distribution. Intergroup differences between the VTE and non‐VTE groups were assessed by a two‐sample *t*‐test for normally distributed continuous variables, a Mann–Whitney *U* test for non‐normally distributed continuous variables, and the χ^2^ test or Fisher exact test for categorical variables. All continuous variables were dichotomized prior to regression modeling. Age and bedridden time were stratified based on clinical guidelines and routine clinical practice. Considering the high‐altitude physiological characteristics and severe inflammatory status of enrolled patients, standard plain laboratory cutoffs could not distinguish VTE risk effectively for PaO_2_/FiO_2_ ratio and D‐dimer; accordingly, their optimal thresholds were determined via ROC curve and the maximum Youden index using in‐hospital VTE as the outcome. Other laboratory indicators were dichotomized referring to conventional clinical laboratory reference ranges. Natural binary clinical event variables were directly incorporated into regression models without cutoff settings. Univariate and multivariate logistic regression analyses were sequentially performed to screen independent VTE risk factors for nomogram construction.

Death was treated as a competitive risk, and 30‐day cumulative incidence curves (point estimates with 95% confidence interval [CI]) were plotted for patients with SCAP. To identify risk factors for VTE, multivariable logistic regression analysis which was based on the factors with significant differences between VTE and non‐VTE groups in univariate analysis and the factors that may be related to dependent variables from the perspective of professional knowledge was performed. The adjusted odds ratio (OR) with 95% CI were reported. Receiver operating characteristic (ROC) curve analysis was performed to evaluate the sensitivity and specificity of risk factors for VTE identification in patients with SCAP. The diagnostic accuracy for screening for VTE of different ROCs curves were compared using the method of DeLong et al. [31]. A nomogram was constructed based on the predictors was drawn in order to enhance the practicability of the prediction model. Calibration was evaluated with calibration plots, which used the bootstrap method of 1000 resampling to show the relationship between the observed frequency and the prediction probability through a graph. In a well‐calibrated model, the prediction should fall on a 45° diagonal. Internal validation of the nomogram model was performed to evaluate its stability and discriminative ability. Ten‐fold cross‐validation was repeated 100 times to calculate the average corrected AUC. Additionally, 1000 bootstrap resamples were applied to obtain the bias‐corrected AUC with 95% CIs, which quantified the optimism‐corrected discriminative performance of the prediction model. Survival curves were plotted using the Kaplan–Meier method and compared between patients with or without VTE using the log‐rank test.

Statistical analyses were performed using SPSS Version 23.0 (Statistical Package for the Social Sciences, Chicago, IL USA) and R software Version 4.3.3. All tests were two‐tailed; *p* < 0.05 was considered statistically significant.

## Results

**FIGURE 1:**
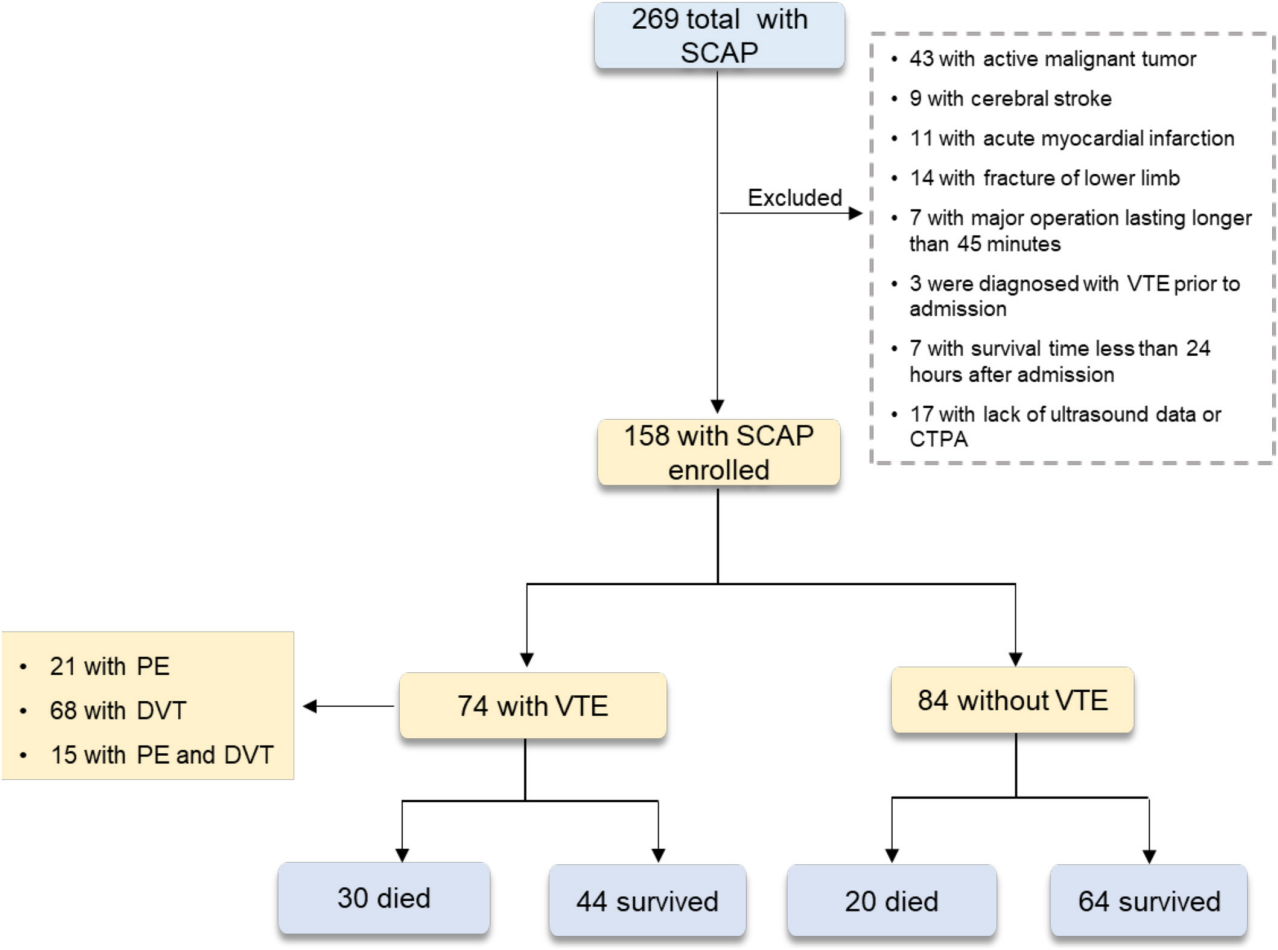
Study flowchart. A total of 158 patients with SCAP were enrolled in this study. Finally, 74 patients developed VTE, including 68 with DVT, 21 with PE, and 15 with both DVT and PE. CTPA, computed tomography pulmonary angiography; DVT, deep vein thrombosis; PE, pulmonary embolism; SCAP, severe community‐acquired pneumonia; VTE, venous thromboembolism.

A total of 158 patients with SCAP were enrolled in the present study. The study flowchart is shown in Figure 1. All patients were prospectively followed up the survival of all patients within 30 days after a diagnosis of SCAP. No patients were excluded or lost during the follow‐up period.

### Imagological Examination for Screening for VTE

**FIGURE 2:**
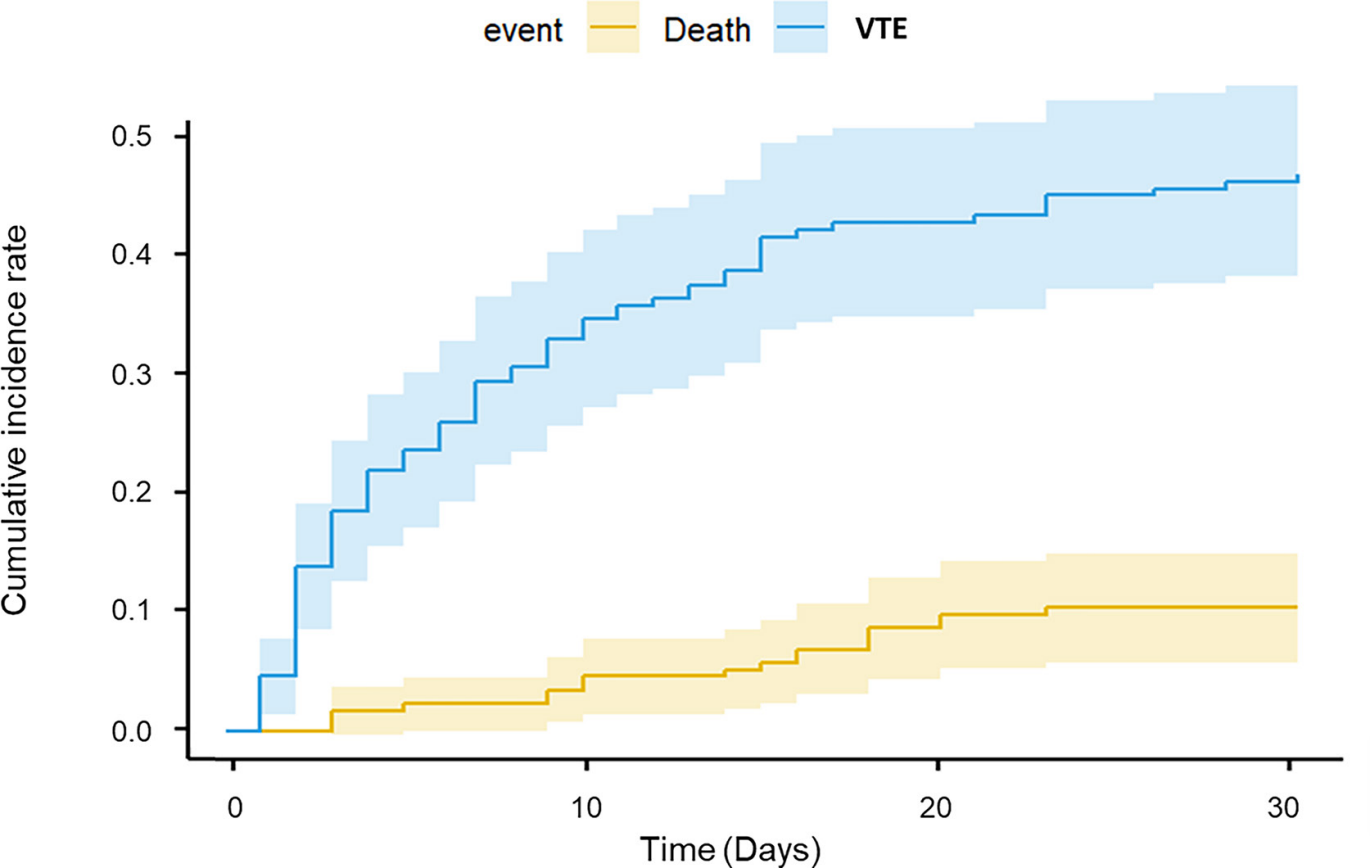
Competitive risk analysis for VTE in patients with SCAP. Taking death as a competitive risk, the 30‐day cumulative incidence curve of VTE was drew for patients with SCAP.

Lower extremity venous compression ultrasound scanning was performed for 158 patients regardless of clinical symptoms of the lower limbs. The median number of ultrasound scans was 2 (range, 1–6). Thirty developed DVT was found, and the other 128 was a negative result at the first ultrasound scan. Subsequently, 86 patients underwent more than one ultrasound scan; among those, 38 developed DVT, and 48 had no DVT with 2 (range, 2–6) ultrasound examinations. There was no difference in the number of ultrasound examinations between these two groups (*p* = 0.818). Finally, of the 158 patients with SCAP, 68 (43.0%) developed DVT, including 14 with proximal DVT and 54 with distal DVT. Among the 68 DVT patients, 57 had no DVT symptoms, with an incidence of asymptomatic DVT 36.1% (57/158), including 13 (8.2%) proximal DVT and 44 (27.8%) distal DVT. For all the 158 patients, the interval from onset to the occurrence of DVT for the 68 patients who developed DVT was 15 (9, 18) days, and the interval from onset to the last ultrasound examination for the 90 cases without DVT was 15 (8, 22) days. There was no difference between the two groups (*p* = 0.799). Among the patients suspected of having PE, 34 underwent CTPA examination, and 21 of these cases were confirmed as PE, with an incidence of PE as 13.3%. In conclusion, 74 (46.8%) patients developed VTE, including 68 with DVT, 21 with PE, and 15 with both DVT and PE. As shown in Figure 2, the 30‐day cumulative incidence of VTE was drew taking death as a competitive risk.

### Demographics and Clinical Characteristics of VTE Versus Non‐VTE Patients With SCAP

Of the 158 SCAP patients enrolled in this study, 152 (96.2) were Tibetan, and there was no difference in the proportion of Tibetans in the VTE group and the non‐VTE group. Among158 patients enrolled, compared patients without VTE, the D‐dimer levels, white blood cell (WBC) counts, neutrophil counts, alanine aminotransferase (ALT) levels, N‐terminal pro B‐type natriuretic peptide (NT‐proBNP) levels, CURB 65 scores, PSI scores, APACHE II scores, SOFA scores, Caprini prediction scores, and Padua prediction scores were higher in patients with VTE (all *p* < 0.05). The PaO_2_/FiO_2_ ratios and adjusted PaO_2_/FiO_2_ ratios were lower, and the bedridden times were longer in patients with VTE (all *p* < 0.05). More patients with VTE had symptoms such as hemoptysis and dyspnea, and more received therapy of intravenous nutrition, CVC and IMV (all *p* < 0.05). There was no difference in age and comorbidities between these two groups (all *p* > 0.05) (Table 1).

**TABLE 1:** Demographics, clinical characteristics, laboratory data, treatments, complications, and prognoses of VTE versus non‐VTE patients with SCAP.

Characteristics	Total (*N* = 158)	VTE (*n* = 74)	Non‐VTE (*n* = 84)	*p*
Age (years)	72 (61, 80)	72 (63, 79)	73 (56, 80)	0.680
BMI (kg/m^2^)	21.9 (19.8, 24.5)	21.6 (19.6, 24.2)	22.2 (19.8, 24.9)	0.360
Male, *n* (%)	95 (60.1)	43 (58.1)	52 (61.9)	0.627
Tibetan nationality, *n* (%)	152 (96.2)	72 (97.3)	80 (95.2)	0.796
Length from onset to admission (days)	7 (3, 10)	7 (3, 12)	7 (3, 10)	0.934
Number of ultrasound scans	2 (1, 2)	2 (1, 2)	2 (1, 2)	0.944
Symptoms and signs of onset				
Fever, *n* (%)	32 (20.3)	11 (14.9)	21 (25.0)	0.114
Cough, *n* (%)	119 (75.3)	56 (75.7)	63 (75.0)	0.922
Hemoptysis, *n* (%)	22 (13.9)	15 (20.3)	7 (8.3)	0.031
Chest pain, *n* (%)	33 (20.9)	15 (20.3)	18 (21.4)	0.858
Dyspnea, *n* (%)	133 (84.2)	68 (91.9)	65 (77.4)	0.013
DVT symptoms, *n* (%)	25 (15.8)	12 (16.2)	13 (15.5)	0.899
Leg pain, *n* (%)	6 (3.8)	2 (2.7)	4 (4.8)	0.796
Edema of low extremities, *n* (%)	20 (12.7)	12 (16.2)	8 (9.5)	0.207
Comorbidities				
History of VTE, *n* (%)	7 (4.4)	5 (6.8)	2 (2.4)	0.344
Current smoker, *n* (%)	18 (11.4)	7 (9.5)	11 (13.1)	0.473
Chronic respiratory disease, *n* (%)	16 (10.1)	9 (12.2)	7 (8.3)	0.426
Hypertension, *n* (%)	64 (40.5)	30 (40.5)	34 (40.5)	0.993
Diabetes mellitus, *n* (%)	12 (7.6)	3 (4.1)	9 (10.7)	0.115
Coronary artery disease, *n* (%)	5 (3.2)	2 (2.7)	3 (3.6)	1.000
Cerebrovascular disease, *n* (%)	10 (6.3)	3 (4.1)	7 (8.3)	0.438
Chronic kidney disease, *n* (%)	9 (5.7)	3 (4.1)	6 (7.1)	0.623
Chronic liver disease, *n* (%)	5 (3.2)	1 (1.4)	4 (4.8)	0.443
Bedridden time (days)	2 (1, 4)	2 (1, 5)	2 (1, 3)	0.025
Scores associated with disease				
CURB‐65 score	3 (2, 3)	3 (2, 3)	2 (2, 3)	0.006
PSI score	103 (91, 117)	104 (91, 123)	100 (90, 113)	0.035
APACHE II score	12 (9, 18)	16 (10, 19)	11 (9, 16)	0.013
SOFA score	4 (3, 7)	5 (3, 7)	4 (2, 6)	0.034
Caprini score	5 (4, 7)	6 (5, 7)	5 (4, 6)	0.008
Padua prediction score	5 (5, 6)	6 (5, 6)	5 (5, 6)	0.003
Laboratory data				
PaO_2_/FiO_2_ ratio (mmHg)	167 (127, 207)	148 (122, 184)	186 (143, 222)	< 0.001
PaO_2_/FiO_2_ ratio adjusted (mmHg)^a^	257 (195, 318)	227 (188, 283)	286 (225, 341)	< 0.001
D‐dimer (μg/mL)	3.0 (1.5, 5.9)	4.1 (2.2, 10.7)	2.1 (1.1, 4.4)	< 0.001
Prothrombin time (seconds)	13.8 (12.6, 15.8)	13.9 (12.7, 16.6)	13.5 (12.4, 15.8)	0.377
Activated partial thromboplastin time (seconds)	38.1 (31.3, 45.9)	38.6 (33.0, 45.9)	36.4 (30.3, 46.0)	0.351
White blood cells (×10^9^/L)	8.2 (5.5, 11.8)	9.1 (6.4, 12.2)	7.4 (4.7, 11.0)	0.013
Neutrophil (×10^9^/L)	6.8 (3.9, 10.3)	7.7 (4.7, 10.9)	6.0 (2.9, 9.2)	0.011
Platelets (×10^9^/L)	159 (99, 225)	166 (107, 227)	152 (85, 222)	0.261
Hemoglobin (g/L)	146 (128, 166)	145 (130, 166)	147 (126, 166)	0.766
C‐reactive protein (mg/L)	71.7 (19.6, 152.8)	69.5 (21.1142.7)	73.0 (18.3160.0)	0.824
Alanine aminotransferase (U/L)	28.1 (16.1, 61.1)	39.5 (18.0, 80.3)	24.9 (14.4, 51.7)	0.033
Fasting blood glucose (mmol/L)	6.7 (5.3, 9.7)	6.5 (5.3, 9.3)	6.8 (5.4, 9.9)	0.453
Serum creatinine (μmol/L)	76.2 (59.7, 106.1)	77.4 (54.5106.1)	74.3 (60.4111.3)	0.833
Cardiac troponin I (ng/mL)	0.09 (0.04, 0.26)	0.09 (0.04, 0.23)	0.10 (0.05, 0.31)	0.515
N‐terminal pro B‐type natriuretic peptide (pg/mL)	1417 (452, 4747)	3217 (601, 5196)	942 (357, 3074)	0.003
Treatments				
VTE prophylaxis, *n* (%)	123 (77.8)	51 (68.9)	72 (85.7)	0.011
LMWH, *n* (%)	104 (65.8)	42 (56.8)	62 (73.8)	0.024
LMWH+ physical prophylaxis, *n* (%)	8 (5.1)	3 (4.1)	5 (6.0)	0.858
Physical prophylaxis only, *n* (%)	19 (12.0)	9 (12.2)	10 (11.9)	0.960
Vasoactive agent therapy, *n* (%)	58 (36.7)	33 (44.6)	25 (29.8)	0.054
Intravenous nutrition, *n* (%)	25 (15.8)	17 (23.0)	8 (9.5)	0.021
Blood transfusion, *n* (%)	22 (13.9)	9 (12.2)	13 (15.5)	0.548
Continuous renal replacement therapy, *n* (%)	6 (3.8)	2 (2.7)	4 (4.8)	0.796
Central venous catheterization, *n* (%)	59 (37.3)	34 (45.9)	25 (29.8)	0.036
Nasal high‐flow oxygen therapy, *n* (%)	103 (65.2)	51 (68.9)	52 (61.9)	0.356
Mechanical ventilation, *n* (%)	69 (43.7)	40 (54.1)	29 (34.5)	0.014
IMV, *n* (%)	51 (32.3)	30 (40.5)	21 (25.0)	0.037
Length of IMV (days)	3 (1, 8)	4 (1, 10)	3 (2, 7)	0.594
Length of IMV ≥ 3 days, *n* (%)	31 (60.8)	19 (63.3)	12 (57.1)	0.656
NIV, *n* (%)	32 (20.3)	18 (24.3)	14 (16.7)	0.232
Prognosis				
Stay in ICU, *n* (%)	109 (69.0)	64 (86.5)	45 (53.6)	< 0.001
Length of ICU stay (days)	10 (4, 13)	11 (6, 16)	8 (3, 12)	0.028
Length of hospital stay (days)	16 (10, 23)	19 (10, 27)	14 (10, 21)	0.022
30‐day mortality, *n* (%)	44 (27.8)	27 (36.5)	17 (20.2)	0.023
In‐hospital mortality, *n* (%)	50 (31.6)	30 (40.5)	20 (23.8)	0.024

*Note:* Data are mean ± SD, median (IQR), or *n* (%). The *p* values comparing VTE and non‐VTE groups were from a two‐sample *t*‐test, Mann–Whitney *U* test, χ^2^ test, or Fisher's exact test. *p* < 0.05 was considered statistically significant.

Abbreviations: APACHE, Acute Physiology and Chronic Health Evaluation; BMI, body mass index; CCI, Charlson comorbidity index; CURB‐65, “confusion, blood urea nitrogen, respiratory rate, blood pressure, age≥65 years”; DVT, deep vein thrombosis; FiO_2_, fraction of inspired oxygen; ICU, intensive care unit; IMV, invasive mechanical ventilation; IQR, interquartile range; LMWH, low molecular weight heparin; NIV, noninvasive mechanical ventilation; PaO_2_, partial pressure of arterial oxygen; PSI, pneumonia severity index; SCAP, severe community‐acquired pneumonia; SD, standard deviation; SOFA, Sequential Organ Failure Assessment; VTE, venous thromboembolism.

^a^ PaO_2_/FiO_2_ × 760/barometric pressure.

Of the 158 patients, 123 (77.8%) were given VTE prophylaxis, for whom 104 (65.8%) received low molecular weight heparin (LMWH; 96 received enoxaparin sodium 40–60 mg once daily, 8 received dalteparin sodium 5000 IU once daily), 19 (12.0%) received physical prevention only, and 8 (5.1%) received combined treatment with LMWH and physical prevention. All the 35 patients who did not receive VTE prophylaxis had anticoagulant therapy contraindications, such as stress ulcers and gastrointestinal bleeding (2 patients), platelet counts less than 50 × 10^9^/L (13 patients), and hemoptysis (20 patients). The incidence of VTE was significantly lower for patients who received VTE prophylaxis than those who did not receive VTE prophylaxis (41.5% [51/123] vs. 65.7% [23/35], respectively, *p* = 0.011). However, for the 123 patients who received VTE prophylaxis, there was no difference in incidence of VTE between patients who received LMWH only (40.6% [39/96]), physical prevention only (47.5% [9/19]), and combined treatment with LMWH and physical prevention (37.5% [3/8]) (*p* = 0.838).

All of the 158 patients received chest computerized tomography (CT) examination; there was no difference in chest CT findings such as consolidation, ground‐glass opacity, mixed density lesions, bilateral pulmonary infiltrates, and pleural effusions between VTE and non‐VTE patients (all *p* > 0.05). A total of 150 (94.9%) patients received echocardiogram examination, with 72 patients in the VTE group and 78 patients in the non‐VTE group. Compared to patients without VTE, those with VTE exhibited higher pulmonary arterial systolic pressure and a higher prevalence of tricuspid regurgitation and PAH (all *p* < 0.05) (Table 2).

**TABLE 2:** Echocardiographic and chest CT findings with VTE versus non‐VTE patients in SCAP.

Variable	Total (*N* = 158)	VTE (*n* = 74)	Non‐VTE (*n* = 84)	*p*
Chest CT findings				
Consolidation, *n* (%)	113 (71.5)	56 (75.7)	57 (67.9)	0.277
Ground‐glass opacity, *n* (%)	114 (72.2)	50 (67.6)	64 (76.2)	0.228
Mixed density lesions, *n* (%)	87 (55.1)	40 (54.1)	47 (56.0)	0.811
Bilateral pulmonary infiltrates, *n* (%)	100 (63.3)	47 (63.5)	53 (63.1)	0.957
Pleural effusion, *n* (%)	81 (51.3)	39 (52.7)	42 (50.0)	0.734
Echocardiographic findings^a^				
LA diameter (mm)	34.7 ± 7.4	35.3 ± 7.6	34.2 ± 7.3	0.379
LVESVI (mL/m^2^)	43.6 ± 7.4	44.0 ± 7.5	43.2 ± 7.3	0.500
LVEDVI (mL/m^2^)	29.7 ± 7.4	30.3 ± 8.1	29.1 ± 6.6	0.304
Simpson biplane EF (%)	60.8 ± 9.4	59.6 ± 11.0	62.0 ± 7.5	0.119
RA diameter (mm)	39.2 ± 9.0	39.9 ± 9.5	38.5 ± 8.5	0.342
RV diameter (mm)	34.4 ± 6.4	34.7 ± 6.9	34.0 ± 6.0	0.508
Tricuspid regurgitation, *n* (%)	126 (84.0)	65 (90.3)	61 (78.2)	0.044
PASP (mmHg)	45.0 (37.0, 55.0)	50.0 (37.0, 59.0)	42.0 (37.0, 51.0)	0.036
PAH, *n* (%)	53 (35.3)	35 (48.6)	18 (23.1)	0.001
Pericardial effusion, *n* (%)	30 (20.0)	13 (18.1)	17 (21.8)	0.567

*Note:* Data are median (IQR) or *n* (%). The *p* values comparing DVT and non‐DVT groups were from Mann–Whitney *U* test or χ^2^ test. *p* < 0.05 was considered statistically significant.

Abbreviations: CT, computerized tomography; EF, ejection fraction; IQR, interquartile range; LA, left atrial; LVEDVI, left ventricular end‐diastolic volume index; LVESVI, left ventricular end‐systolic volume index; PAH, pulmonary artery hypertension; PASP, pulmonary artery systolic pressure; RA, right atrial; RV, right ventricular; SCAP, severe community‐acquired pneumonia; VTE, venous thromboembolism.

^a^ 150 patients received Echocardiography examinations, with 72 patients in the VTE group and 78 patients in the non‐VTE group.

### Independent Risk Factors Associated With VTE for Patients With SCAP

The results of univariate and multivariate logistic regression models are shown in Table 3. To avoid data redundancy, thrombotic prediction scores and disease severity scores were not incorporated into the multivariate regression models. Because the adjusted PaO_2_/FiO_2_ ratio was calculated according to the original PaO_2_/FiO_2_ ratio, we only included the PaO_2_/FiO_2_ ratio rather than its adjusted version to eliminate duplicated data. For the 158 patients with SCAP, the independent contributors to VTE were lower PaO_2_/FiO_2_ ratios (PaO_2_/FiO_2_ ratios ≤ 160 mmHg: OR = 2.354, 95% CI 1.025–5.406, *p* = 0.044), higher D‐dimer level (D‐dimer levels ≥ 3.0 μg/mL: OR = 2.901, 95% CI 1.329–6.334; *p* = 0.008), and longer bedridden time (bedridden times > 3 days: OR = 3.540, 95% CI 1.494–8.391, *p* = 0.004).

**TABLE 3:** Risk factors associated with VTE in patients with SCAP.

Factors	Univariable OR (95% CI)	*p*	Multivariable OR (95% CI)	*p*
Age				
< 65 years	Reference		Reference	
≥ 65 years	1.195 (0.604, 2.364)	0.608	1.615 (0.635, 4.110)	0.314
Hemoptysis				
No	Reference		Reference	
Yes	2.797 (1.072, 7.297)	0.036	3.187 (0.959, 10.588)	0.059
Dyspnea				
No	Reference		Reference	
Yes	3.313 (1.245, 8.816)	0.016	1.578 (0.460, 5.416)	0.469
Bedridden time				
≤ 3 days	Reference		Reference	
> 3 days	3.117 (1.558, 6.234)	0.001	3.540 (1.494, 8.391)	0.004
D‐dimer				
< 3.0 μg/mL	Reference		Reference	
≥ 3.0 μg/mL	3.951 (2.037, 7.665)	< 0.001	2.901 (1.329, 6.334)	0.008
PaO_2_/FiO_2_ ratios				
> 160 mmHg	Reference		Reference	
≤ 160 mmHg	3.727 (1.900, 7.312)	< 0.001	2.354 (1.025, 5.406)	0.044
White blood cells				
< 10.0 × 10^9^/L	Reference		Reference	
≥ 10.0 × 10^9^/L	1.798 (0.933, 3.465)	0.080	1.679 (0.708, 3.981)	0.240
Alanine aminotransferase				
< 40.0 U/L	Reference		Reference	
≥ 40.0 U/L	2.360 (1.228, 4.534)	0.010	1.890 (0.817, 4.374)	0.137
N‐terminal pro B‐type natriuretic peptide				
< 1500 pg/mL	Reference		Reference	
≥ 1500 pg/mL	2.282 (1.205, 4.321)	0.011	1.794 (0.782, 4.117)	0.168
IMV				
No	Reference		Reference	
Yes	2.045 (1.039, 4.028)	0.038	1.062 (0.229, 4.930)	0.939
Central venous catheterization				
No	Reference		Reference	
Yes	2.006 (1.043, 3.858)	0.037	1.830 (0.460, 7.274)	0.391
Vasoactive agent				
No	Reference		Reference	
Yes	2.833 (1.143, 7.023)	0.025	1.406 (0.414, 4.773)	0.585
VTE prophylaxis				
No	Reference		Reference	
Yes	0.370 (0.169, 0.810)	0.013	0.422 (0.151, 1.178)	0.099

*Note:* Multivariable logistic regression was performed in the overall cohort. *p* < 0.05 was considered statistically significant.

Abbreviations: CI, confidence interval; FiO_2_, fraction of inspired oxygen; IMV, invasive mechanical ventilation; OR = odds ratio; PaO_2_, partial pressure of arterial oxygen; SCAP, severe community‐acquired pneumonia; VTE, venous thromboembolism.

### Comparison of Diagnostic Accuracy for Assessing the Risk of VTE of Different ROCs

**FIGURE 3:**
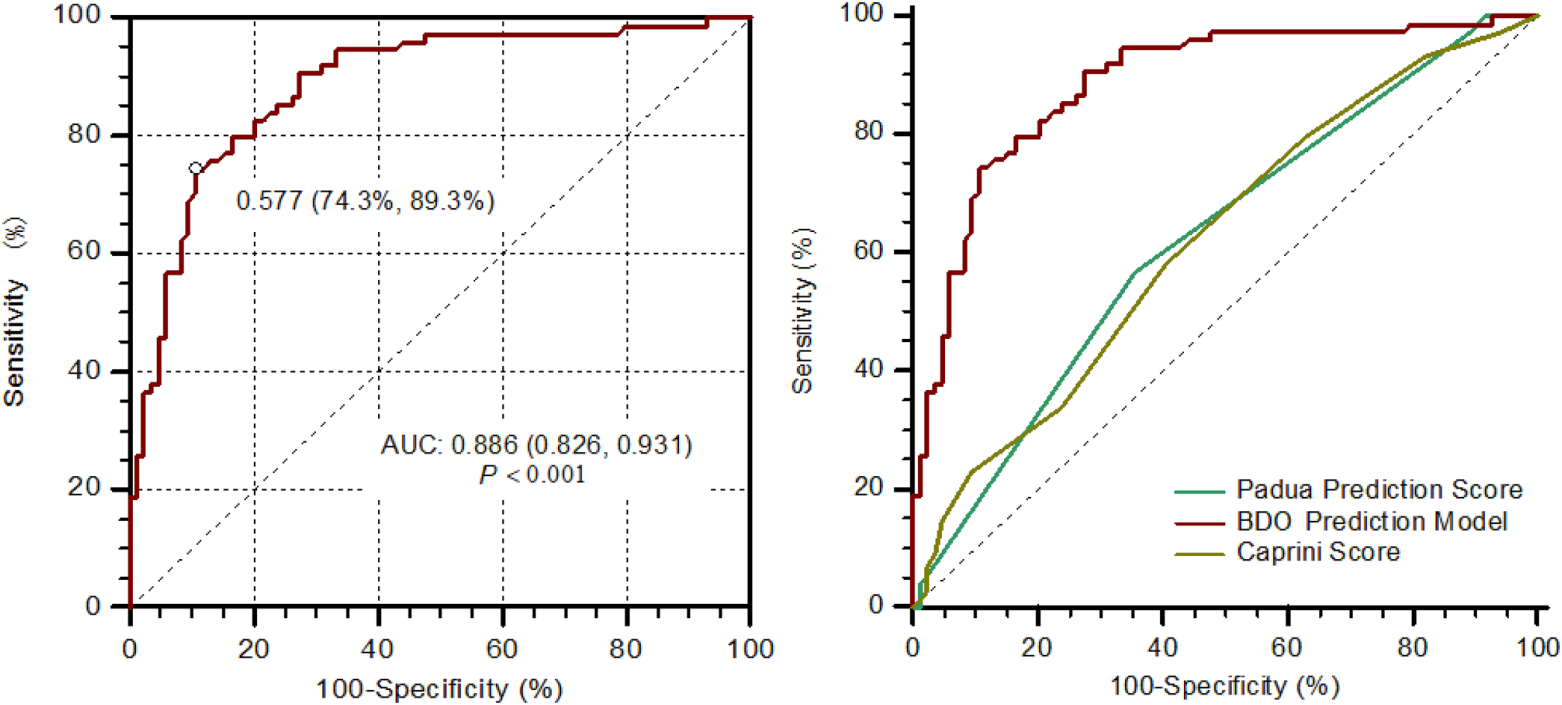
Comparison of diagnostic accuracy for assessing the risk of VTE of different ROCs for patients with SCAP. We selected the risk factors based on the test results of the logistic regression models and proposed a new way of combining forecasting models for assessing the risk of VTE in SCAP patients. The novel prediction model, including bedridden times, D‐dimer levels, and PaO_2_/FiO_2_ ratios, showed a better performance in predicting VTE (AUC = 0.886; 95% CI 0.826–0.931; sensitivity: 74.3%; specificity: 89.3%) than Padua prediction score (AUC = 0.624; *p* < 0.001 for these two models) and Caprini prediction score (AUC = 0.622; *p* < 0.001 for these two models); yet, there was no significant difference between Padua prediction score and Caprini prediction score (*p* = 0.967 for these two curves) when predicting VTE. AUC, area under the curve; CI, confidence interval; FiO_2_, fraction of inspired oxygen; PaO_2_, partial pressure of arterial oxygen; ROC, receiver operating characteristic; SCAP, severe community‐acquired pneumonia; VTE, venous thromboembolism.

We selected the risk factors based on the test results of the logistic regression models and proposed a new way of combining prediction model for assessing the risk of VTE in patients with SCAP. Internal validation was conducted to evaluate the stability and overfitting risk of the model. The average AUC obtained from 10‐fold cross‐validation was 0.891, and the optimism‐corrected AUC via 1000 bootstrap resampling was 0.852 (95% CI 0.823–0.937). The consistent high AUC values across the original cohort and internal validation procedures suggested robust discriminative ability of the nomogram model. The novel prediction model, including PaO_2_/FiO_2_ ratios, D‐dimer levels, and bedridden times showed a better performance in predicting VTE (AUC = 0.886; 95% CI 0.826–0.931; sensitivity: 74.3%; specificity: 89.3%) than Padua prediction score (AUC = 0.624; *p* < 0.001 for these two models) and Caprini prediction score (AUC = 0.622; *p* < 0.001 for these two models) (Figure 3); yet, there was no significant difference between Padua prediction score and Caprini prediction score (*p* = 0.967 for these two curves) when predicting VTE (Figure 3).

### Nomogram for Assessing the Risk of VTE

**FIGURE 4:**
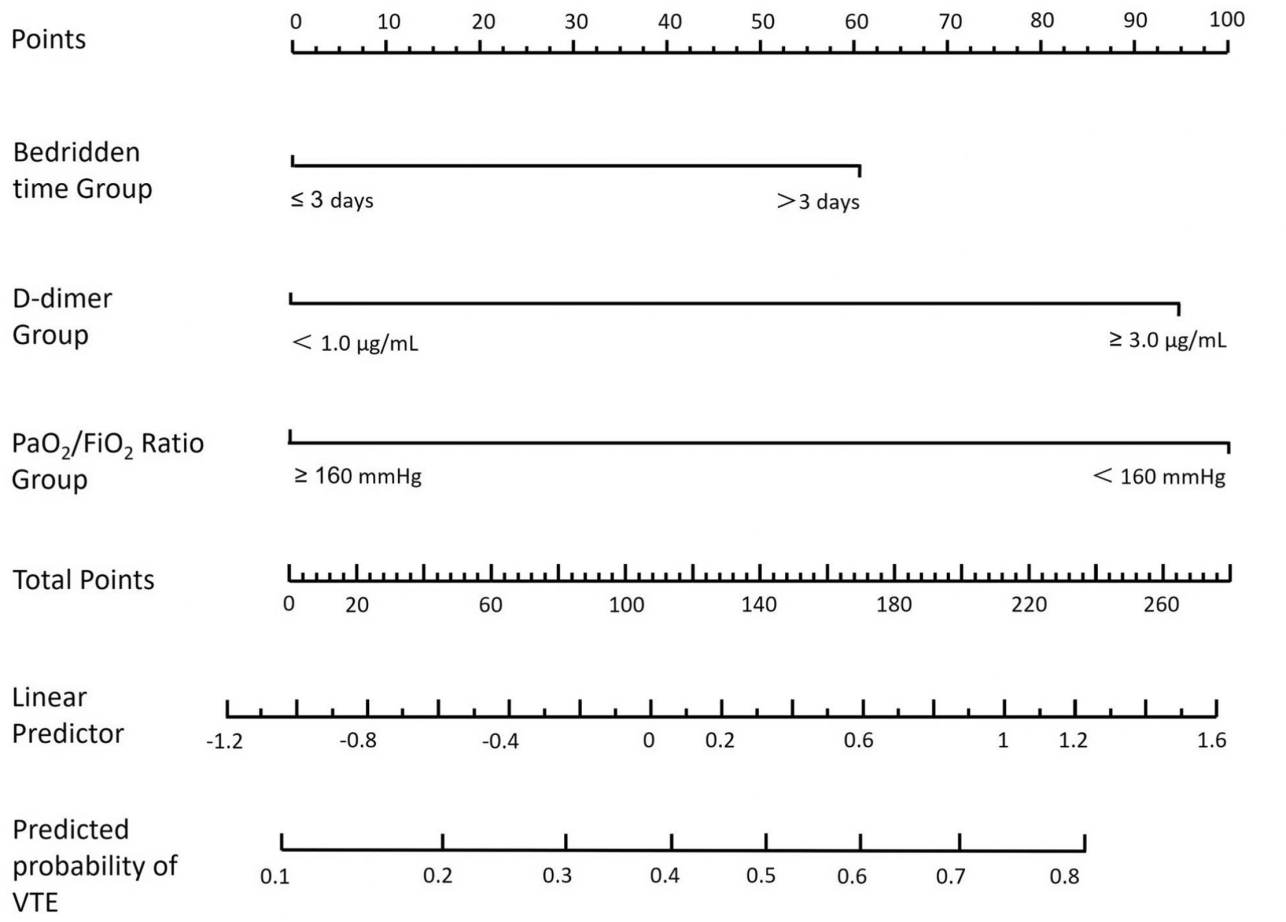
Nomogram for assessing the risk of VTE for patients with SCAP. The nomogram was created based on the selected three predictors. To use the nomogram, draw a vertical line upward from each variable value to obtain the corresponding points. Sum the points from the three predictors to calculate the total points. Then draw a vertical line downward from the total‐points scale to obtain the in‐hospital predicted probability of VTE. FiO_2_, fraction of inspired oxygen; PaO_2_, partial pressure of arterial oxygen; SCAP, severe community‐acquired pneumonia; VTE, venous thromboembolism.

In order to increase the practicability of the prediction model, we created a nomogram based on the selected predictors (Figure 4). There were three prediction variables. The corresponding points were obtained by making a vertical line upward based on the value of each variable. The total points were obtained by adding the points of the three variables. The predicted probability of VTE in hospital was obtained by making a vertical line downward based on the total points. The calibration plots showed good consistency of VTE between the actual observation and the nomogram prediction (Figure S1).

### Prognosis of DVT Patients With SCAP

**FIGURE 5:**
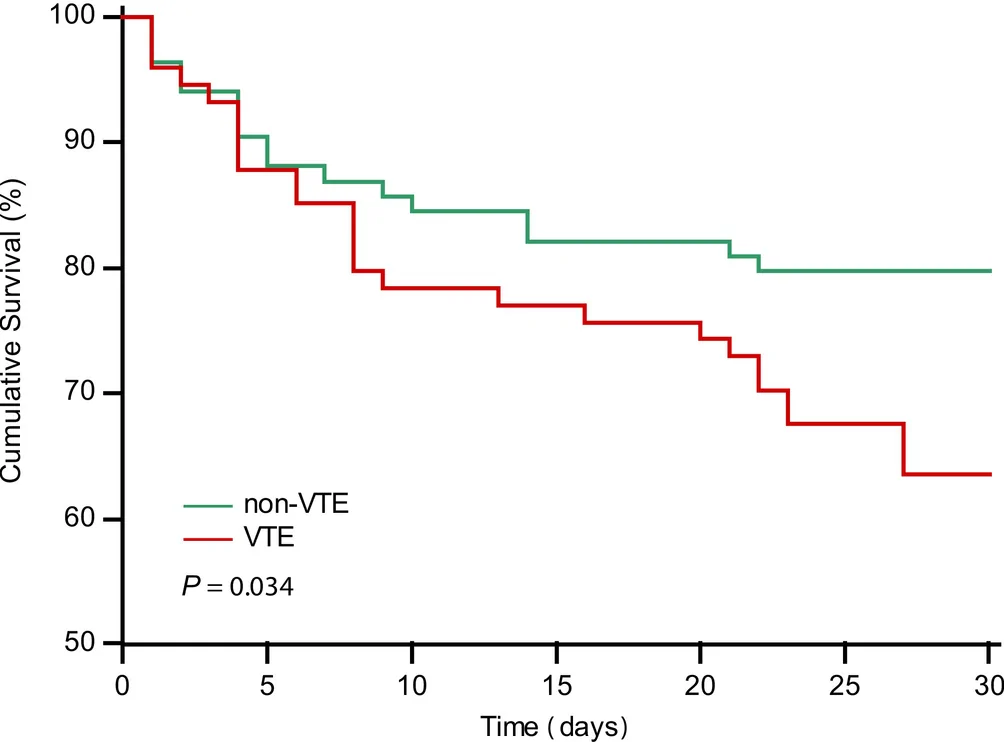
Survival curves for VTE and non‐VTE patients with SCAP. The 30‐day cumulative survival was significantly higher for patients without VTE than patients with VTE (*p* = 0.034; log‐rank test). SCAP, severe community‐acquired pneumonia; VTE, venous thromboembolism.

Among 158 patients with SCAP, compared to patients without VTE, more patients with VTE were admitted to ICU (86.5% [64/74] vs. 53.6% [45/84], respectively; *p* < 0.001). Patients with VTE had longer ICU stays (11 [6, 16] days vs. 8 [3, 12] days, *p* = 0.028) and longer hospital stays (19 [10, 27] days vs. 14 [10, 21] days, *p* = 0.022). Moreover, patients with VTE had a significantly higher 30‐day mortality (36.5% [27/74] vs. 20.2% [17/84], respectively; *p* = 0.023) and in‐hospital mortality (40.5% [30/74] vs. 23.8% [20/84], respectively; *p* = 0.024) (Table 1). The Kaplan–Meier survival curve showed that the 30‐day cumulative survival rate of patients with VTE was lower than that of patients without VTE (*p* = 0.034) (Figure 5).

## Discussion

This single institutional study identified a high prevalence of VTE and associations between VTE and PaO_2_/FiO_2_ ratios, bedridden times, and D‐dimer levels in patients with SCAP in high‐altitude areas.

Of the 158 enrolled SCAP patients in the present cohort, 74 developed VTE events, including 68 cases of DVT, 21 cases of PE, and 15 cases complicated by both DVT and PE. The overall incidence of VTE was 46.8%, which was significantly higher than that observed in critically ill patients in low‐altitude plain regions [14, 32, 33]. The high‐altitude plateau environment confers unique physiological stressors, such as low ambient atmospheric pressure, hypoxia, low temperature, and dehydration [17, 19]. These environmental perturbations may induce a hypercoagulable state by modulating coagulation function, endothelial function, and hemodynamics, thereby increasing the risk of VTE [21, 34]. As early as 2001, Anand et al. [16] reported that individuals residing at high altitudes (> 3000 m) and extremely high altitudes (> 5000 m) had a 30‐fold higher incidence of VTE compared to those in plain areas, an alarming finding. Subsequent studies have also confirmed that both medical and surgical inpatients in high‐altitude regions face a significantly elevated risk of VTE [35, 36, 37]. In recent years, Li et al. investigated the activity of coagulation factors and concentration of transferrin in the plasma of humans residing at different altitudes and mice exposed to high altitude for a short duration [22]. The study found that key high‐altitude factors, such as hypoxia and low temperature, enhance the expression of hypoxia‐inducible factor 1α, upregulate transferrin gene expression, and increase plasma transferrin levels, leading to elevated activities of thrombin and factor XIIa and ultimately VTE. Importantly, treatment with transferrin antibodies or downregulation of transferrin levels significantly improved thromboembolism and pathological damage in mouse models. Additional basic studies also indicated that high altitude and hypoxia can trigger pathological changes such as the coagulation cascade, platelet aggregation, the release of inflammatory factors, endothelial injury, and cellular adhesion by inducing abnormal expression of multiple genes, thereby promoting VTE [18, 20, 34]. In summary, the unique environmental exposure in high‐altitude areas is an important risk factor for VTE.

The risk of VTE increases significantly with the number of risk factors present [38]. In high‐altitude environments, SCAP patients are prone to hypercoagulability and endothelial injury due to severe infections and inflammatory responses. Moreover, restricted mobility secondary to critical illness, combined with endothelial damage caused by CVC implantation and vasoactive drug use, further exacerbates the risk of thrombus formation. These combined factors contribute to the extremely high incidence of VTE in SCAP patients in plateau regions [14, 32, 39].

Within our research, it was discerned that the decreased PaO_2_/FiO_2_ ratios at admission are indicative of an increased risk for VTE among SCAP patients. The high incidence of VTE events at high altitude is closely related to the hypoxia caused by natural environment [19, 20, 21, 22, 35]. Patients with SCAP suffer from severe pulmonary infection and systemic inflammation, resulting in varying degrees of hypoxia aggravation. The PaO_2_/FiO_2_ ratio is an important indicator for assessing pulmonary oxygenation function, and its reduction reflects the extent of pulmonary inflammation and injury. Such damage may involve the pulmonary vascular endothelium, leading to endothelial dysfunction, which in turn causes an imbalance in the local coagulation and anticoagulation systems, promoting thrombus formation [40]. Concurrently, the decrease in the PaO_2_/FiO_2_ ratio may be associated with the disruption of the alveolar‐capillary membrane, increasing the contact between blood and inflammatory mediators in lung and promoting the release of various cytokines, further activating coagulation pathways. Moreover, the reduction in the PaO_2_/FiO_2_ ratio may also be related to the increased demand for oxygen therapy in patients. During the administration of high‐concentration oxygen therapy, the production of oxygen free radicals may increase, causing oxidative stress injury to the vascular endothelium [41] and further promoting the occurrence of venous thrombosis. Therefore, in the high‐altitude environment characterized by hypoxia, the further reduction of PaO_2_/FiO_2_ ratio in SCAP patients may reflect a more severe hypercoagulable state and a tendency to thrombosis.

The present study also identified an association between the elevated D‐dimer levels at admission and the increased incidence of VTE in patients at high‐altitude areas. D‐dimer is a fibrin degradation product encompassing multiple cross‐linked D domains and/or E domains present in the original fibrinogen molecule, whose generation is only theoretically possible when hemostasis and fibrinolysis pathways are concomitantly activated. Measurement of plasma D‐dimer level has now become a basic element in the diagnosis or exclusion and prognostication of VTE when incorporated into validated clinical algorithms. Tsaplin et al. conducted a cohort study involving 168 patients with coronavirus disease 2019 (COVID‐19) to compare the predictive efficacy of the revised Caprini scoring system for VTE risk. The latter, which includes elevated D‐dimer levels, demonstrated superior accuracy in predicting VTE risk among the studied population [42]. Jacobson and colleagues' research has revealed that high‐altitude air travel induces a hypercoagulable state in healthy volunteers, with a concomitant increase in D‐dimer levels [43]. Sun and Yang et al. have further discovered that among inpatients in the Qinghai province of the Qinghai–Tibet Plateau, an elevated D‐dimer level is significantly associated with an increased incidence of VTE [23, 44]. Thus, it can be seen that, similar to what is observed in plain areas, monitoring D‐dimer levels in high‐altitude regions is equally indispensable for assessing the tendency towards hypercoagulability and predicting the occurrence of VTE events. It is a well‐documented phenomenon that patients afflicted with CAP, irrespective of whether the etiology is viral or bacterial, frequently present with increased D‐dimer levels and an augmented risk of VTE [45, 46], a concern that is particularly pronounced in those SCAP. In our study, we observed a significant elevation in D‐dimer levels among SCAP patients, and the degree of this elevation was found to be significantly correlated with the occurrence of VTE. This finding also underscores the potential value of D‐dimer levels as a predictive marker for VTE risk in patients with SCAP at high‐altitude areas, providing a robust basis for early identification and intervention in clinical settings.

Aligning with previous literature and existing VTE prediction models [26, 27], prolonged bed rest was also identified as a risk factor for the development of VTE in this study. Patients who have been bedridden for more than 3 days exhibited a threefold increase in the risk of VTE. As one of the significant contributing factors to the occurrence of VTE [47, 48], prolonged bed rest and immobilization have been incorporated into various VTE risk assessment models and authoritative guidelines [26, 27, 38]. Mechanistically, prolonged bed rest leads to a reduction in physical activity, which in turn slows the return of blood from the lower extremities, causing stasis and thereby increasing the risk of thrombus formation. Furthermore, an extended period of bed rest often indicates a more severe condition in the patient, potentially accompanied by a more serious inflammatory response and a hypercoagulable state, which in turn raises the risk of developing VTE. In conclusion, in high‐altitude environments where thrombotic events were inherently prothrombotic, prolonged bed rest or immobilization in critically ill patients further contributes to the occurrence of VTE.

Inconsistent with previous studies and validated VTE prediction models [26, 27], the present study did not identify an association between advanced age and the elevated risk of VTE in patients with SCAP. This discrepancy may be attributable to the relatively limited sample size of the present cohort. Alternatively, it could be that in the highly prothrombotic environment of high‐altitude areas, the impact of aging on the occurrence of VTE attenuates, rendering age a non‐dominant risk factor in this specific patient.

Unexpectedly, our multivariate analysis failed to uncover a significant correlation between VTE prophylactic measures and the reduced incidence of VTE. This may be attributed to the fact that SCAP patients in high‐altitude settings harbor a multitude of thrombotic risk factors, which collectively drive the incidence of VTE to an alarmingly high level. The preventive measures endorsed by current guidelines seem to fall short of meeting the urgent needs of this particular population for VTE prevention. This finding also outlines a clear roadmap for future research, which is to meticulously optimize the prophylactic measures and conduct a comprehensive and in‐depth evaluation of their preventive efficacy in this specific group, in an effort to explore more precise and effective approaches for VTE prevention in patients with SCAP at high altitudes.

Collectively, among SCAP patients in high‐altitude areas, the occurrence of VTE is closely associated with a decrease in PaO_2_/FiO_2_ ratio, an increase in D‐dimer levels, and a prolonged bedridden period. When these three indicators are combined, their accuracy in predicting VTE is significantly enhanced, surpassing the commonly used Caprini and Padua predictive scores in current clinical practice [26, 27]. The likely reason is that the unique natural environment of high‐altitude areas leads to a strong prothrombotic tendency. Moreover, the severe systemic inflammatory response, vascular endothelial injury, and immobilization associated with SCAP further exacerbate the hypercoagulable state, causing the risk of thrombosis to markedly elevate thrombotic susceptibility. Nevertheless, conventional clinical models, such as the Caprini and Padua predictive scores [26, 27], are designed for general hospitalized populations in plain areas and cannot meet the assessment needs of specific diseases in the special environment of high altitudes. In particular, the correlation between the PaO_2_/FiO_2_ ratio and VTE in the new model more specifically highlights the prominent pathological changes of severe pneumonia in the special high‐altitude environment, thus enabling more accurate and effective prediction of VTE occurrence.

Similar to prior investigations [45, 46, 49], the present study demonstrated a significant association between VTE and adverse clinical prognosis in SCAP patients. Notably, the 30‐day cumulative survival was markedly lower among patients with concurrent VTE compared to those without VTE. Univariate analysis revealed that patients with VTE exhibited elevated WBC counts, neutrophil counts, and D‐dimer levels compared to patients without VTE, indicating a potential increase in inflammatory response and subsequent exacerbation of coagulation and fibrinolysis dysfunction [50, 51, 52]. Additionally, individuals in the VTE cohort presented prolonged immobilization, higher NT‐proBNP levels, lower PaO_2_/FiO_2_ ratios, and elevated severity scores such as CURB65 scores, PSI scores, APACHE II scores, and SOFA scores, suggesting that these patients may represent a more severe subset of SCAP. In addition, SCAP is a serious condition, usually accompanied by severe hypoxia. In the case of PE, hypoxia, abnormal pulmonary hemodynamics and right ventricular load are further aggravated, and in severe cases, death is caused. Although these findings are plausible given that our population consists of critically ill patients with poor prognosis, the high incidence of VTE and its associated worse clinical outcomes underscore the importance of screening for VTE‐related risk factors in such patients. It is imperative to explore specific and optimal VTE prevention strategies to improve the prognosis of these individuals.

In this study, we conducted a prospective observational study of SCAP patients in high‐altitude areas, which, despite its valuable insights, was not without limitations. Firstly, the study included only 158 patients, a relatively small sample size. The small sample size could have reduced the statistical power to detect such associations, thereby potentially overlooking important risk factors and, concurrently, restricted our capacity to conduct model validation. Secondly, the assessment of bedridden time was mainly based on clinical records and physicians' comprehensive judgment, which carried a certain degree of subjectivity and might introduce potential measurement bias. Although immobilization is a well‐established risk factor for VTE, the definition and recording of bedridden duration may vary among different medical staff, which could exert a certain impact on the performance of the prediction model. Thirdly, the critical nature of SCAP posed significant challenges. Some patients with a high clinical suspicion of PE were too ill to undergo further diagnostic procedures such as CTPA. This constraint likely led to an underestimation of the true incidence of PE or VTE, as the most severely affected patients could not be fully evaluated for these conditions. Fourthly, our study did not include a comparison group of patients with the same condition from plain regions. Such a comparison would have provided valuable context and allowed for a more nuanced understanding of the unique impact of high‐altitude conditions on SCAP and VTE risk. Lastly, the guideline‐recommended VTE prophylactic measures implemented in our study appeared to be insufficient in preventing VTE events. This finding suggests that the current preventive strategies may need to be optimized, but specific measures for optimizing prevention are currently lacking. If resources permit, further controlled studies are warranted to explore more effective VTE prevention strategies. In conclusion, although our study contributes to the understanding of VTE in patients with SCAP in high‐altitude settings, these limitations highlight the need for larger, more comprehensive studies that can address these issues and provide more definitive guidance on VTE prevention in this specific patient cohort.

## Conclusions

The incidence of VTE is extremely high in patients with SCAP in high‐altitude areas and is associated with lower PaO_2_/FiO_2_ ratios, higher D‐dimer levels, and longer bedridden times. The new prediction model including three factors above outperforms traditional scoring systems in VTE risk assessment, providing a more accurate predictive tool for clinical use in specific settings. Because the 30‐day survival rate of patients with VTE is significantly reduced, early identification of high‐risk groups and aggressive intervention are critical. Future studies should further optimize preventive strategies to reduce the risk of VTE in SCAP patients in high‐altitude areas.

## Author Contributions

Yuntao Zhang and Yanyan Zhang designed the study, collected clinical data, analyzed the data, and wrote the manuscript. Miao Lu, Basang Xiao, Jianghe Jia, Xingya Shi, and Xiwangmu Yi designed the study and wrote the manuscript. Na Cui and Yuanhua Yang helped manage the research, performed the statistical analyses, and revised the paper. Yuntao Zhang and Yanyan Zhang contributed equally to this article and share first authorship. Na Cui and Yuanhua Yang contributed equally to this article and share corresponding authorship. All authors read and approved the final manuscript.

## Funding

This work was supported by the Natural Science Foundation of Xizang Autonomous Region (Grant No. XZ2024ZR‐ZY033 [Z] to Na Cui), the National Key Clinical Specialty Construction Project (Respiratory and Critical Care Medicine Department of Lhasa People's Hospital), and Lhasa Municipal Clinical Research Center for High‐Altitude Health.

## Ethics Statement

The study was approved by the ethics committees of the Lhasa People's Hospital (SYLL2124057) and was conducted in accordance with the 1964 Helsinki Declaration and its later amendments or comparable ethical standards.

## Consent

Informed consent had been obtained from all patients at the time of hospital admission.

## Conflicts of Interest

The authors declare no conflicts of interest.

## Supporting information


**Figure S1:** Supporting Information.

## Data Availability

All data analyzed during the study are presented in the main manuscript and Supporting Information. In addition, the anonymous dataset is available from the corresponding author upon reasonable request.
