# Supplementary material for: Venous Thromboembolism in Severe Community‐Acquired Pneumonia at High‐Altitude Areas: Prevalence, Risk and Outcome
Source: Clin Respir J. 2026 Sep 10;20(9):e70230. doi: 10.1111/crj.70230 (PMC13562917; doi:10.1111/crj.70230)
Supplement: Supplementary file 1 [file CRJ-20-e70230-s001.pdf]

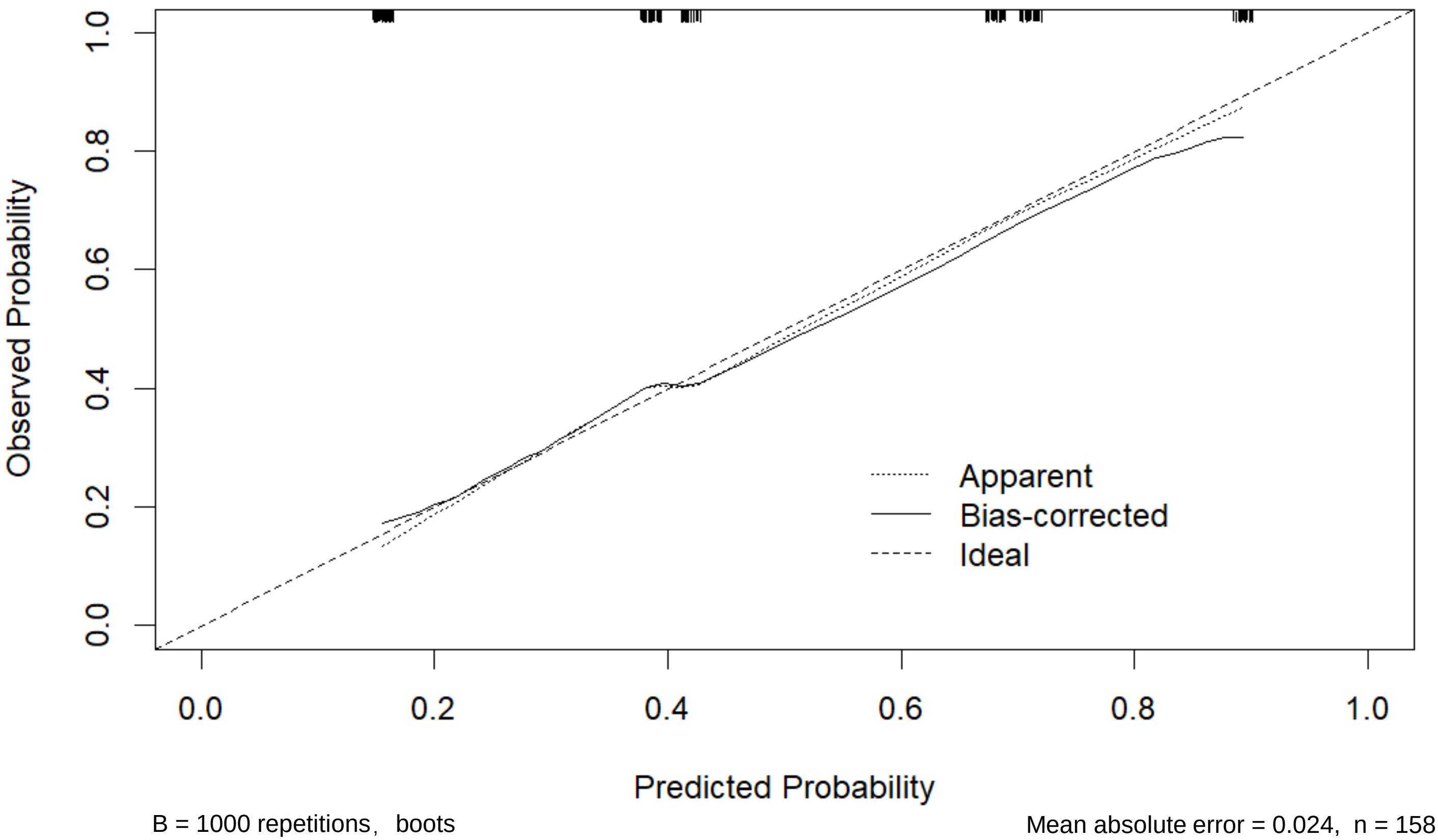

1.0
0.8
0.6
0.4
0.2
0.0
Observed Probability
Apparent
Bias-corrected
Ideal
0.0
0.2
0.4
0.6
0.8
1.0
Predicted Probability
B = 1000 repetitions，boots
Mean absolute error = 0.024，n = 158
